# Fish Oil Suppresses Cell Growth and Metastatic Potential by Regulating PTEN and NF-κB Signaling in Colorectal Cancer

**DOI:** 10.1371/journal.pone.0084627

**Published:** 2014-01-08

**Authors:** Shevali Kansal, Archana Bhatnagar, Navneet Agnihotri

**Affiliations:** Department of Biochemistry, Panjab University, Chandigarh, India; Henry Ford Health System, United States of America

## Abstract

Homeostasis in eukaryotic tissues is tightly regulated by an intricate balance of the prosurvival and antisurvival signals. The tumor suppressor PTEN (phosphatase and tensin homolog deleted on chromosome 10), a dual-specificity phosphatase, plays a functional role in cell cycle arrest and apoptosis. NF-κB and its downstream regulators (such as VEGF) play a central role in prevention of apoptosis, promotion of inflammation and tumor growth. Therefore, we thought to estimate the expression of PTEN, Poly-ADP-ribose polymerase (PARP), NF-κBp50, NF-κBp65 and VEGF to evaluate the effect of supplementation of fish oil on apoptotic and inflammatory signaling in colon carcinoma. Male wistar rats in Group I received purified diet while Group II and III received modified diet supplemented with FO∶CO(1∶1)&FO∶CO(2.5∶1) respectively. These were further subdivided into controls receiving ethylenediamine-tetra acetic-acid and treated groups received dimethylhydrazine-dihydrochloride (DMH)/week for 4 weeks. Animals sacrificed 48 hours after last injection constituted initiation phase and that sacrificed after 16 weeks constituted post-initiation phase. We have analysed expression of PTEN, NF-κBp50, NF-κBp65 by flowcytometer and nuclear localization of NF-κB by immunofluorescence. PARP and VEGF were assessed by immunohistochemistry. In the initiation phase, animals receiving DMH have shown increased % of apoptotic cells, PTEN, PARP, NF-κBp50, NF-κBp65 and VEGF however in post-initiation phase no significant alteration in apoptosis with decreased PTEN and increased PARP, NF-κBp50, NF-κBp65 and VEGF were observed as compared to control animals. On treatment with both ratios of fish oil in both the phases, augmentation in % of apoptotic cells, decreased PTEN, PARP, NF-κBp50, NF-κBp65 and VEGF were documented with respect to DMH treated animals with effect being more exerted with higher ration in post-initiation phase. Hence, fish oil activates apoptosis, diminishes DNA damage and inhibits inflammatory signalling in a dose and time dependent manner so as to inhibit progression of colon cancer.

## Introduction

Epidemiological reports have shown that the incidence of cancer is increasing worldwide [1]. Cancer is the disease in which there is a deregulation of cell proliferation and cell death. In cancerous cells, endogenous signal transduction pathways get disturbed to redirect the cellular decisions from differentiation and apoptosis to proliferation and, later, invasion [2]. Phosphatidylinositol 3-kinase (PI3K) signaling pathway has a crucial role in promoting cell survival by inhibiting the apoptosis. PI3K converts the plasma membrane lipid phosphatidylinositol 4,5-bisphosphate (PIP_2_) to phosphatidylinositol 3,4,5-triphosphate (PIP_3_), which then recruits proteins that contain a pleckstrin homology (PH) domain to cellular membranes [3] such as phosphoinositol dependent kinase-1 (PDK1) and Akt. Activated Akt is the predominant and essential mediator for the regulation of apoptosis and proliferation by targeting different downstream substrates: IκB kinase, Bcl-2, cytochrome c and others [4], [5]. Activity of the PI3K/Akt pathway is negatively regulated by the phosphatase and tensin homolog deleted on chromosome 10 (PTEN). PTEN dephosphorylates the 3′OH group and converts PIP_3_ into PIP_2_, leading to the activation of apoptosis and hence functions as a tumour suppressor [6]. The reports have been shown that the expression of *PTEN* has been transcriptionally suppressed through the activation of NF-κB. The NF-κB consists of transactivation subunit RelA/p65 and the DNA-binding subunits p50 and p52, which are processed from the precursor's p105 and p100 respectively [7]. NF-κB is sequestered in the cytoplasm by the inhibitor IκB to prevent its transcriptional activation in unstimulated conditions. The inflammatory cytokines cause the phosphorylation of IκB, which in turn releases NF-κB, which subsequently translocates into the nucleus and affect the expression of target genes having key roles in the inhibition of apoptosis, promotion of tumour growth, and activation of inflammatory responses which further promotes the metastasis by increasing the expression of vasucular endothelial growth factor (VEGF) [8]. The covalent modification of proteins by Poly(ADP-ribosyl)ation is an immediate and dramatic biochemical response to DNA damage. It is a ubiquitous protein modification found in mammalian cells that modulates many cellular responses, including DNA repair. Poly-ADP-ribose polymerase (PARP) catalyzes the polymerization of ADP-ribose units from donor NAD^+^ molecules on target proteins, resulting in the attachment of linear or branched polymers [9]. PARP exhibit pleiotropic cellular functions ranging from maintenance of genomic stability and chromatin remodelling to regulation of cell death, thereby rendering the PARP homologues as a promising targets in cancer therapy [10].

Epidemiological reports have shown that colorectal cancer (CRC) is the third most common cancer in men (after prostate cancer and lung cancer) and women (after breast cancer and lung cancer) [1]. Experimental studies have shown that a variety of factors are linked with the development of CRC [11]. The previous experiments conducted in our laboratory have demonstrated that the administration of fish oil (n-3 PUFA)/corn oil (n-6 PUFA) in 2.5/1 ratio has a better efficacy as compared to 1/1 for the chemoprevention of experimentally induced colorectal cancer [12]. Another study has shown that chemopreventive action of different ratios of fish oil and corn oil might be mediated through Ras signaling pathway [13]. One of the another study from our lab has demonstrated that fish oil (FO) and corn oil (CO) in 2.5∶1 ratio altered the mitochondrial membrane parameters, ROS, and Ca^2+^ in such a way so as to increase apoptosis in both phases, whereas FO∶CO in 1∶1 ratio enhanced apoptosis only in post-initiation phase [14]. It has been earlier shown that EPA and DHA increased the level of PTEN which in turn inhibited the transcription of anti-apoptotic genes, hence demonstrated the beneficial effects of fish oil on breast tumour cell growth [15]. Therefore, the present study was conducted to understand the role of different ratios of fish oil and corn oil on apoptotic pathways and metastatic potential mediated by PTEN and NF-κB in experimentally induced colon cancer.

## Materials and Methods

### Chemicals

*N,N′*-Dimethylhydrazine dihydrochloride (DMH), paraformaledhyde (PFA), propidium iodide (PI), Hoechst 33342 (H342) and bovine serum albumin (BSA) were obtained from Sigma Chemical Company, St. Louis, USA. The monoclonal antibody against PTEN was procured from genescript, Piscataway, NJ, USA. The monoclonal antibodies against PARP, VEGF, NF-κB p50 and NF-κB p65 were purchased from Santacruz, CA, USA and BD biosciences, MD, USA respectively. Fluorescein isothiocyanate (FITC)-conjugated goat anti-mouse IgG_1_ was purchased from Bangalore Genei, Bangalore, India. Maxepa fish oil [containing 180 mg Eicosapentaenoic acid (EPA) and 120 mg Docosahexaenoic acid (DHA)/ml] was obtained from Merck Chemicals Limited, Goa, India and Corn oil containing 58.8% linoleic acid, 26.4% oleic, 1.3% linolenic and 12.8% saturated fatty acid was procured from Sigma Chemical Company, St. Louis, USA. The mineral mixture (Agrimin) was obtained from Virbac Animal health India Pvt. Ltd., Mumbai, India. All other chemicals used in the study were of analytical grade.

### Animals and Diet

Male Wistar rats weighing 100–200 gm were obtained from and housed in the Central Animal House, Panjab University, Chandigarh. The experimental protocols were approved by the “Institutional Animal Ethics Committee, Panjab University, Chandigarh” (**Ref. no.** 1-12/IAEC 3/9/2009) and conducted according to guidelines of the “Indian National Science Academy” for the use and care of experimental animals. The animals were housed in polypropylene cages in the animal house and were acclimatized before being used in the experimental study. Water was given *ad libitum*. After one week of acclimatization, animals were randomly divided into the different groups and were fed experimental diet for four weeks. The composition of experimental diet has been given in Table 1. The diets were prepared on the basis of the American Institute of Nutrition standard reference diet AIN-76A [16]. The composition of all experimental diets was adjusted so that animals in all the groups would consume the same amount of calories [12].

**Table 1:** Effect of fish oil and corn oil on the PTEN levels.

Groups	Net MFI(MFI of cells treated with Ab - MFI of cells only)	
	Initiation phase	Post-initiation phase
Control group	131.67±14.51	127.60±17.12
DMH treated	194.17±08.93***	72.17±08.23***
FO∶CO(1∶1)+EDTA	152.00±10.91	132.04±09.07
FO∶CO(1∶1)+DMH	164.17±12.38###	174.33±17.58###
FO∶CO(2.5∶1)+EDTA	139.83±08.88	130.40±10.57
FO∶CO(2.5∶1)+DMH	164.40±05.98###	220.83±09.51### ^,^ $$$

The results are expressed as Mean±S.D. for n = 8.

p<0.001 wrt control group,

^###^ p<0.001 wrt DMH treated,

p<0.001 wrt FO+CO(1∶1)+DMH.

### Experimental Design

**Figure 1:**
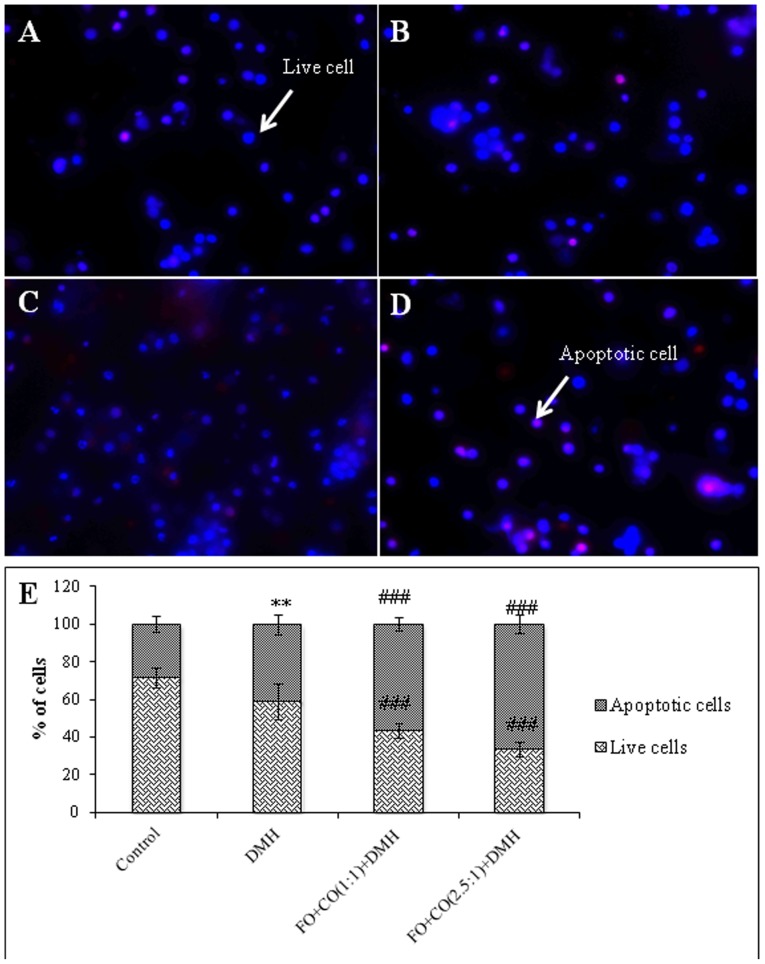
Effect of different ratios of fish oil and corn oil on % of apoptotic cells in the initiation phase of experimentally induced colon cancer. The isolated colonocytes were incubated with Hoechst 342 (H342) dye and PI. Photomicrographs were acquired using fluorescent microscope (Nikon Eclipse 80*i*) A- control group, B- DMH treated, C) FO+CO(1∶1)+DMH, D) FO+CO(2.5∶1)+DMH. Dark blue colour represents the normal live cells, faint blue or pink represents apoptotic cells (Magnification 400×). E) Graphical representation of % of live and apoptotic cells in different groups. The results are expressed as Mean±S.D. for n = 4 (^**p^<0.01 wrt control, ^###^p<0.001 wrt DMH).

The experimental design is represented in Fig. 1. Male Wistar rats (N = 96) were equally divided into the following experimental groups:

***Control group)***- These animals received purified diet and a weekly intraperitoneal injection of 1 mM ethylenediamine tetra-acetic acid (EDTA), pH 6.5, for a period of 4 weeks.

***DMH treated*** - The animals of this group were given purified diet along with a weekly intraperitoneal injection of DMH (20 mg/kg body weight) for 4 weeks.

***FO+CO (1∶1)+EDTA*** The animals received a modified diet supplemented with 1∶1 ratio of FO and CO. A weekly intraperitoneal injection of EDTA was also given for a period of 4 weeks.

***FO+CO (1∶1)+DMH*** A modified diet supplemented with 1∶1 ratio of FO and CO was given to the animals of this group and a weekly intraperitoneal injection of DMH for a period of 4 weeks.

***FO+CO (2.5∶1)+EDTA*** The animals of this group were given modified diet supplemented with FO+CO in the ratio of 2.5∶1 and received a weekly intraperitoneal injection of EDTA for a period of 4 weeks.

***FO+CO (2.5∶1)+DMH*** A modified diet supplemented with FO+CO in the ratio of 2.5∶1 was given to the animals of this group and a weekly intraperitoneal injection of DMH for a period of 4 weeks.

Each group was further subdivided for studies on initiation and post initiation phase and the animals were equally distributed between the two phases. The animals which were sacrificed 48 h after the last EDTA/DMH injections constituted the initiation phase [17] and the animals kept for 12 weeks after the treatment regimen constituted the post-initiation phase study. All the animals were sacrificed by cervical dislocation.

### Isolation of the colonocytes

The colonocytes were isolated by the method of Sanders [18]. The entire colon was cut longitudinally to expose lumen and placed in warm Ca^2+^ and Mg^2+^ free-Hank's buffered salt solution (HBSS), 30 mmol/l EDTA, 5 mmol/l dithiothreitol (DTT), 0.1% BSA (bovine serum albumin). After a 15 min shaking incubation at 37°C, the mucosal side was gently scraped to remove the intact crypts. The isolated cells were then centrifuged at 2200 rpm and washed twice in warm HBSS containing 1.3 mM CaCl_2_, 1 mM MgSO_4_ and 0.1% BSA. The cells were counted using a haemocytometer and their viability was checked by trypan blue exclusion method [19].

### Estimation of live and apoptotic cells

The percentage of live and apoptotic cells were assessed by immunofluorescence. Isolated colonocytes (1–2×10^6^) were resuspended in 1 ml of PBS and incubated with 10 µl of Hoechst 342 (H342) dye (1 mM) at 37°C for 1 hour. The cells were washed twice and resuspended in PBS. Then the cells were incubated with 10 µl of PI (1 mg/ml) at 37°C for 10 min. The cells were again washed and resuspended in PBS. Photomicrographs were acquired using the fluorescent microscope (Nikon Eclipse 80*i*) and analyzed using Northern Eclipse imaging Elements-D (NIS-D) software. Photomicrographs of cells with H342 and PI were merged. In merged photomicrographs, cells with pink colour cells and cells with dark blue colour on periphery represent apoptotic cells having condensed/fragmented DNA whereas fainted blue all over were considered to be the normal cells.

### Analysis of PTEN, NF-κB p50 and NF-κB p65 by flowcytometer

The intracellular proteins were estimated by flowcytometer. The colonocytes were fixed in 4% paraformaldehyde for 20 minutes at room temperature. After washing with PBS twice, the colonocytes were permeabilized with 100% ice cold methanol (added drop wise) and left for 15 min at −20°C. The cells were washed again in cold PBS twice. Approximately 1×10^6^ cells were added to a FACS tube, resuspended in saponin buffer (PBS containing 0.1% saponin and 2% BSA) and incubated for 30 min at 4°C. The different aliquots of colonocytes were then incubated with diluted PTEN, NF-κB p50 and NF-κB p65 (1∶100) monoclonal antibodies for 30 min at room temperature and then washed once with saponin buffer. The colonocytes were then incubated with diluted FITC conjugated secondary antibody for 45 min at room temperature. The cells were washed once with saponin buffer and then with PBS. The cells were resuspended in PBS. The acquisition from each sample was conducted on FACS Canto (BD Biosciences, US) and the collected data was analyzed using the BD FACS Diva software. The subsequent controls for PTEN, NF-κB p50 and NF-κB p65 were also run simultaneously. The results of PTEN, NF-κB p50 and NF-κB p65 were represented as the mean of Net MFI (MFI of cells treated with Ab - MFI of cells only).

### Localization of NF-κB p50 and NF-κB p65 by immunofluorescence

The isolated fixed colonocytes were washed with ice cold PBS twice. The cells were air dried on glass slides (VWR Scientific, Thane, Maharashtra, India) and allowed to adhere for 10 min at room temperature. The cells were permeabilized with 0.5% Triton X-100 and non-specific binding was blocked using 2% (w/v) BSA in PBS for 30 min at room temperature, prior to incubation with diluted monoclonal antibody against NF-κB p50 (1∶50) and NF-κB p65 (1∶50) for 1 hour at room temperature. Samples were washed with PBS, incubated with diluted FITC-conjugated anti-mouse IgG_1_ for 2 hour at room temperature and again washed with PBS. The sections were counterstained with PI for 10 min at room temperature and then washed with PBS. The sections were mounted and sealed with the clear nail paint. The images were acquired using a fluorescent microscope (Nikon Eclipse 80*i*) and analyzed with Northern Eclipse imaging Elements-D (NIS-D) software. Photomicrographs of cells with NF-κB p50/p65 and PI were merged. After merging the photomicrographs, green colour indicates the expression of NF-κB p50/p65 in the cytoplasm of the cell, red colour represents nuclear staining with PI and yellow colour indicates the localization of NF-κB p50/p65 in the nucleus of the cell.

### Immunohistochemical estimation of VEGF and PARP

The tissue sections (2–3 µm thick) were mounted on poly-L-lysine coated slides. The slides were heated at 65°C before deparaffinization in xylene. The slides were rehydrated with serial alcohol solutions (100%, 90%, 70%, 50%, 30%). Endogenous peroxidase activity was quenched by incubating the slides with 3% H_2_O_2_ (in methanol) for 20 min at 4°C. The sections were blocked using 2% BSA in PBS for 30 min at room temperature. Antigen retrieval was done with retrieval buffer (pH 6.0) by using the microwave (microwaved 5 min for VEGF and 5 min ×2 for PARP). The slides were allowed to cool for 20 minutes. Following antigen retrieval, the sections were incubated with VEGF (1∶100) and PARP (1∶50) antibodies for overnight at 4°C in a humid chamber. The slides were washed in PBS and followed by incubation of 2 hour with HRP-conjugated anti mouse antibody (1∶100) for VEGF and HRP-conjugated anti rabbit antibody (1∶100) for PARP at 37°C in a humid chamber. The slides were visualized using DAB and H_2_O_2_. Sections were then counterstained with haematoxylin for 2 min, followed by rinsing in deionized H_2_O. Slides were dehydrated and mounted with DPX for analysis. Images were acquired and analyzed using the Nikon Eclipse 80*i* microscope (Japan) and Northern Eclipse imaging Elements-D (NIS-D) software.

### Statistical Analysis

The results were expressed as Mean ± S.D. The differences between the groups were assessed by ANOVA after ascertaining normality by Q-Q plot. The software used for statistical analysis was SPSS 18.0 software package for windows. The statistical significance was determined by one-way ANOVA with Bonferroni multiple comparison post hoc tests, and differences were considered significant for p<0.05.

## Results

### Effect of supplementation of fish oil on apoptosis/necrosis in the experimentally induced colon cancer

**Figure 2:**
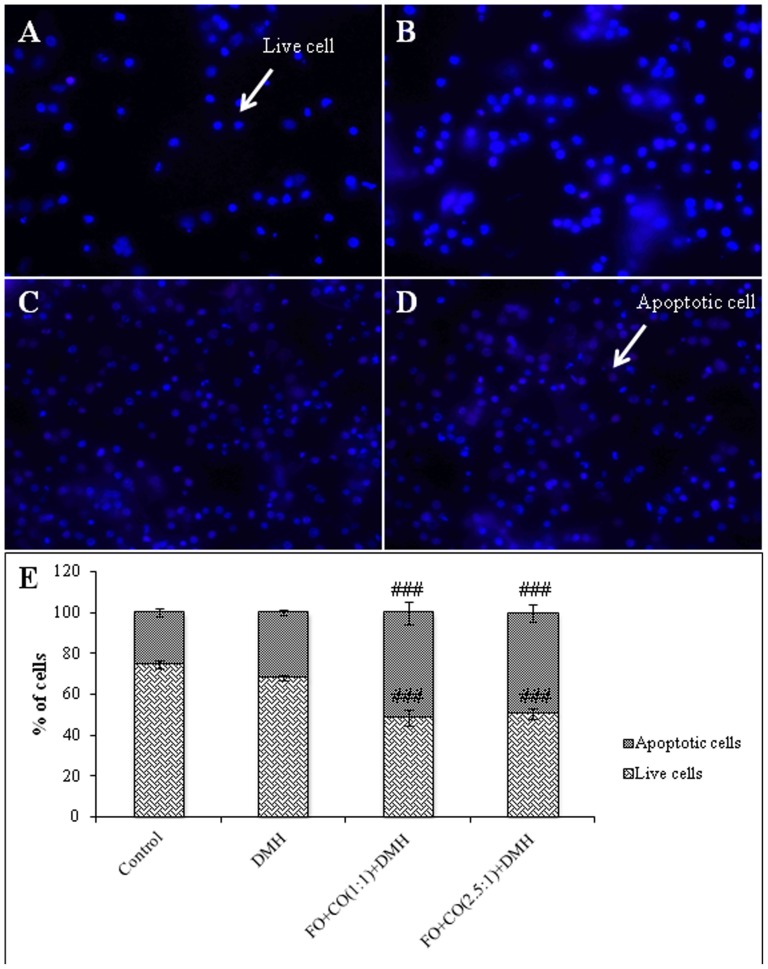
Alterations in the % of apoptotic cells on administration of both the ratios of fish oil and corn oil in post-initiation phase. The isolated colonocytes were incubated with Hoechst 342 (H342) dye and PI. Photomicrographs were acquired using fluorescent microscope (Nikon Eclipse 80*i*) A- control group, B- DMH treated, C) FO+CO(1∶1)+DMH, D) FO+CO(2.5∶1)+DMH. Dark blue colour represents the normal live cells, faint blue or pink represents apoptotic cells (Magnification 400×). E) Graphical representation of % of live and apoptotic cells in different groups. The results are expressed as Mean±S.D. for n = 4 (^###^p<0.001 wrt DMH).

In the present study, % of apoptotic cells was calculated using immunofluorescence and the results are depicted in fig. 1 and 2. In the initiation phase, animals receiving DMH have shown a significant increase in % of apoptotic cells as compared to the control animals (Figure 1). However, on treatment with FO+CO(1∶1)+DMH and FO+CO(2.5∶1)+DMH, a significant decrease in % of live cells and a significant augmentation in % of apoptotic cells were observed as compared to DMH treated animals. It has been observed that in post-initiation phase, on treatment with DMH, there was no significant alteration in the % of apoptotic cells as compared to the control animals (Figure 2). On receiving both the ratios, of fish oil and corn oil, it has been demonstrated that a significant decline in % of live cells and a significant enhancement in % of apoptotic cells as compared to DMH treated animals.

### Alteration in the expression of PTEN on treatment with different ratios of fish oil

After evaluating alterations in % of apoptotic cells on receiving these PUFAs, we then thought to examine the expression of regulator of apoptosis. The tumour suppressor PTEN (phosphatase and tensin homolog) is the most important regulator apoptotic signalling pathway. Therefore, in current study, the expression of PTEN was estimated and the results of PTEN are given in Table 1. In initiation phase, the flowcytometric data has depicted that expression of PTEN was elevated significantly in DMH treated animals as compared to control animals whereas on treating the animals with different ratios of FO+CO, PTEN was increased considerably with respect to control animals but the increase was less as compared to DMH treated animals. The post-initiation phase data has demonstrated that PTEN expression was decreased significantly on treatment with DMH with respect to control animals. However, on treatment with both the ratios of fish oil and corn oil, the PTEN expression was increased considerably. The increase was more significant with FO+CO(2.5∶1)+DMH in the post-initiation phase.

### Effect of supplementation of fish oil and corn oil on PARP activity

**Figure 3:**
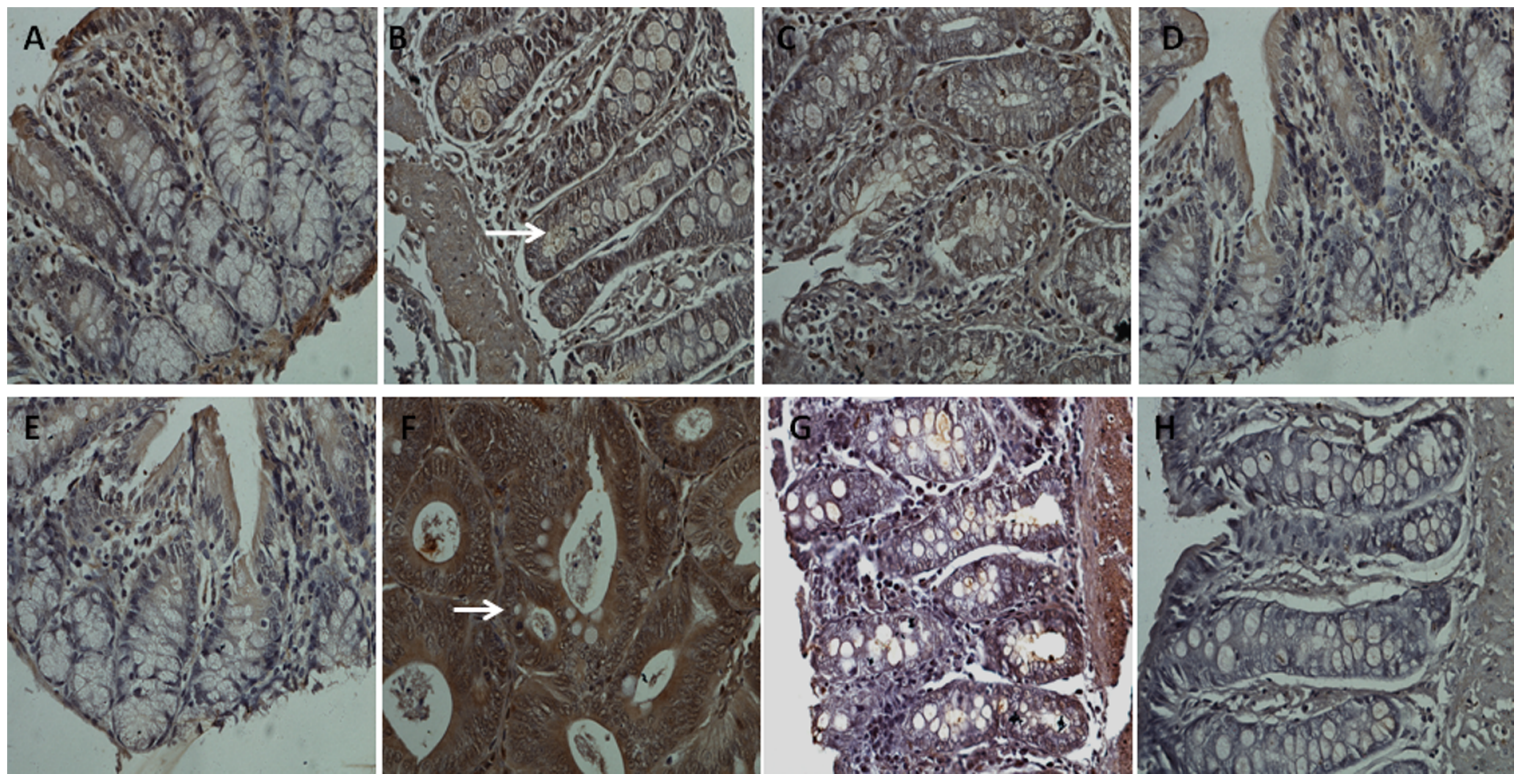
Inhibition of PARP activity on receiving different ratios of fish oil and corn oil in experimentally induced colon cancer. Immunohistochemical staining of PARP in formalin fixed paraffin embedded sections of colon tissue. A–D depicts the representative photomicrographs of initiation phase and E–H represents the post-initiation phase (“arrow” depicts the expression of PARP). (A and E) Control, (B and F) DMH treated, (C and G) FO+CO(1∶1)+DMH, (D and H) FO+CO(2.5∶1)+DMH (Magnification 400X).

PARP is an abundant DNA-binding enzyme that detects the DNA strand breaks. PARP enzyme plays an important role in different cellular functions, thereby rendering PARP as a promising target in cancer therapy [10]. Therefore, in the current study, activity of PARP was analyzed to examine the effect of different ratios of fish oil and corn oil on DNA repair enzyme. The results of immunohistochemical staining of PARP are shown in fig. 3. The colonic mucosa of control animals has depicted very moderate or weak expression of PARP. On treatment with DMH, PARP expression was augmented in both the phases as compared to control animals. The cells were strongly positive for the PARP in the post-initiation phase as compared to the initiation phase. However, on receiving the different ratios of fish oil and corn oil, the expression of PARP was declined as compared to DMH treated animals with the effect being pronounced with FO+CO(2.5∶1)+DMH in the post-initiation phase.

### Downregulation of NF-κB subunits on supplementation of fish oil

The activation of NF-κB pathway is a crucial event in inflammation-induced tumour growth and progression [20]. NF-κB is a ubiquitous transcriptional factor that plays a pivotal role in cell survival and cell death [21]. NF-κB is present in the cytosol in a bound form with IκB. Phosphorylation of serine residues of IκB proteins by IκB kinases (IKKs), targets the IκB for proteasomal degradation. The free NF-κB subunits (p50 and p65) are transported to the nucleus as a dimer and target the promoters which lead to the activation of genes that are involved in cell proliferation, invasion and cell death. NF-κB is negatively regulated by PTEN and further targets many anti-apoptotic genes such as Bcl-2 and Bcl-XL [15]. Therefore, after evaluating the expression of PTEN, we then thought to analyze the expression as well as localization of both subunits of NF-κB in experimentally induced colon cancer.

### Expression and Localization of NF-κB p65

The expression of NF-κB p65 has been measured by flowcytometer and the localization was examined using fluorescence microscope. The flow-cytometric results of initiation phase depicted that expression of NF-κB p65 has been significantly increased on treatment with DMH as compared to the control animals. On treatment with FO+CO(1∶1)+DMH, NF-κB p65 was further augmented significantly however, on receiving FO+CO(2.5∶1)+DMH, expression of NF-κB p65 was declined significantly in comparison to DMH treated animals (Table 2). The post-initiation phase data has revealed that on giving DMH, NF-κB p65 expression was increased significantly as compared to control animals. On treatment with both the ratios of FO+CO+DMH, the expression was decreased significantly with respect to DMH treated group. The decrease was more significant with FO+CO(2.5∶1)+DMH as compared to FO+CO(1∶1)+DMH.

**Table 2:** Effect of fish oil and corn oil on NF- κB p65 expression.

Group	Net MFI(MFI of cells treated with Ab - MFI of cells only)	
	Initiation phase	Post-initiation phase
**Control group**	37.25±04.78	55.20±06.30
**DMH treated**	79.75±12.44***	160.00±16.18***
**FO∶CO(1∶1)+EDTA**	39.80±02.49	54.00±15.08
**FO∶CO(1∶1)+DMH**	113.75±10.96###	106.40±11.08###
**FO∶CO(2.5∶1)+EDTA**	34.50±06.60	51.40±09.31
**FO∶CO(2.5∶1)+DMH**	62.75±09.14### ^,^ $$$	75.80±08.52### ^,^ $$$

The results are expressed as Mean±S.D. for n = 8.

p<0.001 wrt control group,

^###^ p<0.001 wrt DMH treated,

p<0.001 wrt FO+CO(1∶1)+DMH.

**Figure 4:**
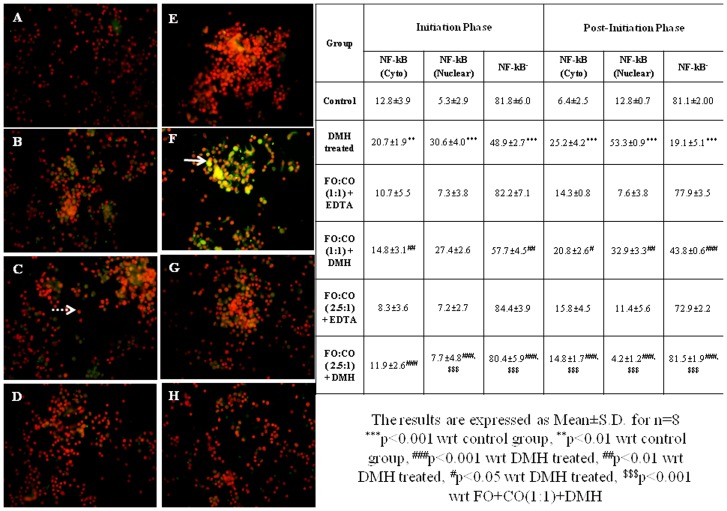
Effect of supplementation of fish oil on nuclear localization of NF-κBp65 in initiation and post-initiation phase. The isolated fixed colonocytes were permeabilized and incubated with monoclonal antibody against NF-κB p65 and corresponding FITC conjugated secondary antibody The sections were counterstained with PI to visualise nuclear localisation. Red fluorescence represents the nuclear staining by PI with no expression of NF-κB (depicted by “dotted arrow”) and green fluorescence indicates the expression of NF-κB p65. Yellow colour indicates the localisation of NF-κB p65 from cytoplasm to nucleus (represented by “arrow”). A–D depicts the initiation phase and E–H represents the post-initiation phase. (A and E) Control, (B and F) DMH treated, (C and G) FO+CO(1∶1)+DMH, (D and H) FO+CO(2.5∶1)+DMH (Magnification 400X).

As the activated NF-κB subunits translocate from cytoplasm to nucleus, therefore, in the present study, nuclear localization of NF-κB was also evaluated. The results of localization of NF-κB p65 are summarized in fig. 4. The immunofluorescence data has revealed that on treatment with DMH, the % of cells having NF-κB p65 in the nucleus and cytoplasm was increased significantly in both phases as compared to control animals (fig. 4B & 4F). However, on treatment with different ratios of fish oil and corn oil, localization of NF-κB p65 from cytoplasm to the nucleus was reduced with the effect being more marked with FO+CO(2.5∶1)+DMH in the post-initiation phase.

### Expression and Localization of NF-κB p50

Flow-cytometric analysis has shown that on treatment with carcinogen, expression of NF-κB p50 was increased significantly (p<0.001) in both the phases as compared to control animals (Table 3). However, on treatment with FO+CO(1∶1)+DMH and FO+CO(2.5∶1)+DMH, the expression of NF-κB p50 was decreased significantly (p<0.001) in comparison to DMH treated animals in both the initiation as well as post-initiation phase.

**Table 3:** Effect of fish oil and corn oil on NF- κB p50 expression.

Group	Net MFI(MFI of cells treated with Ab - MFI of cells only)	
	Initiation phase	Post-initiation phase
**Control group**	53.80±08.01	57.20±07.95
**DMH treated**	109.80±06.97***	200.40±14.91***
**FO∶CO(1∶1)+EDTA**	49.20±06.41	62.00±04.47
**FO∶CO(1∶1)+DMH**	82.25±06.70###	103.00±14.71###
**FO∶CO(2.5∶1)+EDTA**	48.25±11.29	58.80±08.70
**FO∶CO(2.5∶1)+DMH**	73.50±10.08###	68.80±06.97### ^,^ $$$

The results are expressed as Mean±S.D. for n = 8.

p<0.001 wrt control group,

^###^ p<0.001 wrt DMH treated,

p<0.001 wrt FO+CO(1∶1)+DMH.

**Figure 5:**
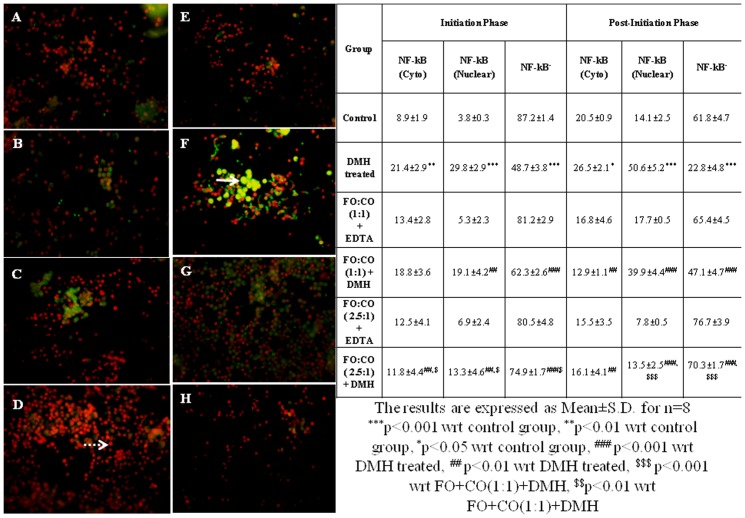
Inhibition of nuclear localization of NF-κBp50 on receiving the different ratios of fish oil in both the phases of experimentally induced colon cancer. The isolated fixed colonocytes were permeabilized and incubated with monoclonal antibody against NF-κB p65 and corresponding FITC conjugated secondary antibody The sections were counterstained with PI to visualise nuclear localisation. Red fluorescence represents the nuclear staining by PI with no expression of NF-κB (depicted by “dotted arrow”) and green fluorescence indicates the expression of NF-κB p65. Yellow colour indicates the localisation of NF-κB p65 from cytoplasm to nucleus (represented by “arrow”). A–D depicts the initiation phase and E–H represents the post-initiation phase. (A and E) Control, (B and F) DMH treated, (C and G) FO+CO(1∶1)+DMH, (D and H) FO+CO(2.5∶1)+DMH (Magnification 400X).

In the present study, localization of NF-κB p50 from cytoplasm to nucleus was done by double labeling. Immunofluorescence data has revealed that on treatment with DMH, the % of cells with NF-κB p50^+^ in nucleus and cytoplasm were augmented significantly in both the phases as compared to control animals (fig. 5B & 5F). However, on treatment with different ratios of fish oil and corn oil, the localization of NF-κB p50 from cytoplasm to the nucleus was declined with the effect being more pronounced with FO+CO(2.5∶1)+DMH in the post-initiation phase.

### Effect of fish oil on VEGF in both the initiation and post-initiation phase of colon cancer

**Figure 6:**
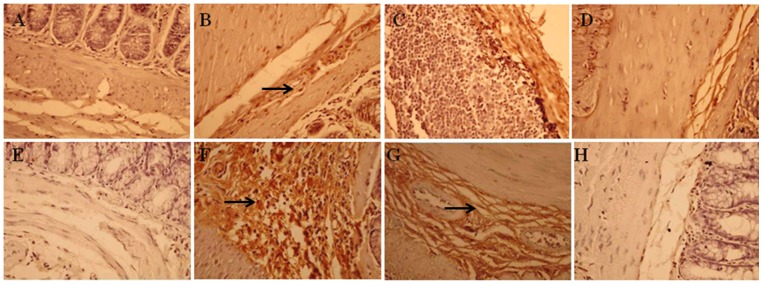
Alterations in VEGF expression on administration of both the ratios of fish oil and corn oil. The expression of VEGF was analyzed using immunohistochemistry in formalin fixed paraffin embedded sections of colon tissue. A–D depicts the representative photomicrographs of initiation phase and E–H represents the post-initiation phase (→ depicts the expression of VEGF). (A and E) Control, (B and F) DMH treated, (C and G) FO+CO(1∶1)+DMH, (D and H) FO+CO(2.5∶1)+DMH (Magnification 400X).

Angiogenesis is an essential component of normal embryonic development, requiring complex interactions between endothelial cells and cells in the surrounding tissue. Signaling by VEGF and their receptors VEGFRs play key roles in these interactions [22]. VEGF binds to these receptors and stimulates endothelial cell proliferation, migration and survival. Therefore in the present study, the effect of supplementation of fish oil and corn oil on the expression of VEGF was estimated. The results of immunohistochemical staining of VEGF for the current study are depicted in fig. 6. The colonic mucosa of control animals has depicted a weak expression of VEGF in the endothelial cells. On treatment with DMH, the expression of VEGF was elevated in the endothelial cells of the colon in both the phases as compared to control animals. The increase in the expression of VEGF was more pronounced in the post-initiation phase as compared to the initiation phase. On receiving the FO+CO(1∶1)+DMH, there was no significant difference in the expression of VEGF in the initiation phase as compared to the DMH treated animals, however, treatment with FO+CO(1∶1)+DMH in the post-initiation phase, the expression of VEGF was decreased. On receiving FO+CO(2.5∶1)+DMH, the expression of VEGF was decreased in both the phases as compared to DMH treated animals.

## Discussion

It has been demonstrated by the earlier reports that DHA and EPA, two active components of fish oil, prevent the transcription of NF-κB dependent genes and increases the expression of PTEN in pancreatic, breast and colon cancer cells. The previous studies conducted in our laboratory have demonstrated that FO+CO(2.5∶1) has better chemopreventive efficacy as compared to FO+CO(1∶1) in experimentally induced colon cancer [12]. Therefore, the current study was conducted to understand the mechanism of chemopreventive action of these dietary PUFAs on the apoptotic pathways. The results of our study have proved that addition of fish oil and corn oil in the diet exerts differential chemopreventive effect by increasing the apoptosis which might be mediated through alteration in the expression of PTEN and NF-κB signaling.

A major regulator of apoptosis is the PTEN pathway. PTEN antagonizes PI3K activity by converting PIP3 back to PIP2, and thereby, reducing the cellular pool of PIP3 [23], [24]. The loss of function of tumor suppressor gene PTEN, is the most common genetic aberration in the cancer [25]. Apart from its functions in regulating the PI3K/Akt pathway, loss of function of PTEN also causes genetic instability [26]. Poly (ADP-ribose) polymerase (PARP) comprises a family of proteins that are involved in DNA repair by utilizing the base excision repair (BER) pathway [27]. Hence, in the current study, the % of apoptotic cells and the expression of PTEN were analyzed in order to understand their contribution towards the apoptotic pathway in the chemoprevention mediated by different ratios of fish oil and corn oil in experimentally induced colon cancer. The expression of PARP-1, as an indicator of DNA damage or genomic instability, was also studied in both the initiation as well as post-initiation phase.

In the current study, on treatment with DMH, an increase in percentage apoptotic cells and PTEN activity was observed in the initiation phase. A previous report from the lab had also documented an increase in apoptosis in the initiation phase on treatment with DMH [12]. However, in post-initiation phase, on treatment with DMH, the expression of PTEN was decreased, whereas no alteration was observed in the apoptotic levels with respect to control animals. DMH treatment has been reported to increase apoptosis in initiation phase followed by a drop after 18 weeks [28]. It has been hypothesised that this increase in apoptosis after treatment with a chemical carcinogen might be due to the destruction of cells bearing DNA damage [29]. An increase in the expression of PARP-1 in DMH treated animals in the current study also corroborated the above hypothesis. An escalation in the expression of DNA repair enzyme is an adaptive change to the increase in DNA damage on treatment with DMH. PARP has been reported to be activated in response to different cellular stresses such as DNA repair, recombination, stalled replication forks, gene rearrangements, as well as by oxidative stress [30]. PARP-1 has been found to be overexpressed in a variety of cancers and its expression has been associated with overall prognosis in cancer [31].

An increase observed in PTEN in initiation phase also supports our results of elevated levels of apoptosis. PTEN has been documented to be the initial event for the development of cancer and was, therefore, altered in the initiation stage itself. An increase in PTEN together with the enhanced apoptosis suggests that in this stage, the cells are trying to combat the initial changes caused by the metabolites of DMH and hence, prevent the development of cancer by destroying the damaged cells [28]. During post-initiation phase, a decrease in expression in PTEN represents the advanced stage of cancer as has been reported earlier [32]. Therefore, it suggests that there is the progression of experimentally induced colon cancer in this stage.

On treatment with both the ratios of FO+CO(1∶1) and FO+CO(2.5∶1) alongwith DMH, the percentage of cells undergoing apoptosis and necrosis were significantly elevated in both the phases as compared to DMH treated animals with the effect being more pronounced with the higher ratio.

In initiation phase, both the ratios of fish oil and corn oil resulted in an elevation in PTEN expression in relation to control animals only; however, it was lower than that observed in DMH treated animals. In post-initiation phase, both the ratios led to amplification in PTEN expression in comparison to both the control and DMH treated animals with the effect being more marked in FO+CO(2.5∶1)+DMH group. An augmentation in PTEN and apoptosis reflects the initial changes of defence against chemically induced colon carcinogenesis [33]. However, an increase in PTEN in the post-initiation phase, on treatment with both the ratios of fish oil and corn oil suggests the modulation of the levels of PTEN by fish oil and corn oil. Hence, the results of the current study suggest that both the ratios of fish oil and corn oil exert chemopreventive effect by increasing apoptosis which might be mediated through PTEN pathway. It has been documented earlier also that fish oil exerts its chemopreventive effect in breast cancer through increased expression of PTEN [15], [34]. The results on apoptosis can also be explained by the fact that, the expression of PARP diminished on treatment with both the ratios of FO and CO in both the phases. The decrease was more significant with FO+CO(2.5∶1)+DMH in the post-initiation phase. A reduction in PARP expression on treatment with both the ratios is suggestive of a decreased DNA damage or genomic stability on supplementation of fish oil and corn oil. Another reason for inactivation of PARP can be its cleavage by activated caspases into 89 and 24 kDa subunits which separates the DNA-binding domain from the catalytic domains. [35], [36].

The activation of NF-κB pathway is a crucial event in inflammation-induced tumour growth and progression [20]. NF-κB is a ubiquitous transcriptional factor that plays a pivotal role in cell survival and cell death [21]. NF-κB is present in the cytosol in a bound form with IκB. Phosphorylation of serine residues of IκB proteins by IκB kinases (IKKs), targets the IκB for proteasomal degradation. The free NF-κB subunits (p50 and p65) are transported to the nucleus and target the promoter genes that are involved in cell proliferation, invasion and cell death [37]–[39]. In addition to inhibition of apoptosis, activated NF-*κ*B may control cell cycle progression by regulating the expression of important cell cycle regulatory proteins such as cyclin D1 and cyclin dependent kinase 2 (CDK2), further contributing to the tumour growth [40]. NF-κB also negatively regulates proapoptotic tumor suppressor PTEN expression which promotes cell survival. The constitutive nuclear activation of NF-*κ*B could also play a major role in the endogenous expression of chemokines, interleukins IL-1, IL-6 and VEGF hence, lead to the pathogenesis of cancer [41], [42]. VEGF is a predominant pro-angiogenic factor, which upon binding to its receptor, stimulates the expression of important factors including anti-apoptotic proteins, cell adhesion molecules and matrix metalloproteinases (MMPs), thereby, promoting the migration and survival of cells [43]. Therefore, in the current study, the expression and localization of two subunits of NF-κB i.e. p50 and p65 and its downstream target i.e. VEGF were analyzed to assess the chemopreventive action of different ratios of fish oil and corn oil in experimentally induced colon cancer.

The expression of both the subunits of NF-κB i.e. p65 and p50 was increased on treatment with DMH in both the phases. The results were further substantiated by immunofluorescence which not only showed an elevation in the expression of these subunits but also their nuclear localization, thereby suggesting an activation of NF-κB. Moreover, the percentage of cells expressing p65 and p50 in the nucleus was approximately equal which suggests the presence of NF-κB dimer in the nucleus. The increased NF-κB nuclear immunostaining of p65 and p50 have also been documented earlier in several tumours such as breast, colon, ovarian, pancreatic, bladder and cervical carcinomas [44], [45]. The results of the current study have also shown that the expression of VEGF was increased in both the phases on treatment with carcinogen. The elevated expression of NF-κB and its target genes, VEGF, would result in invasion and metastasis which suggests the progression of experimentally induced colon cancer [41], [46].

In the initiation phase, on treatment with FO+CO(1∶1)+DMH, the expression of p65 has been increased whereas that of p50 decreased. However, immunofluorescence studies have revealed that the nuclear localization of both p65 and p50 was significantly declined in both the phases on treatment with FO+CO(1∶1)+DMH as compared to DMH treated animals. This suggests that even though the expression of p65 was increased, there was inhibition in nuclear localization of p65 i.e. NF-*κ*B is not activated. Treatment with higher ratio of fish oil and corn oil, FO+CO(2.5∶1)+DMH, results in the attenuation in p65 expression and nuclear localization in both the phases in comparison to FO+CO(1∶1)+DMH which exhibited this effect only in the initiation phase. The expression of VEGF was also decreased on treatment with both the ratios of fish oil and corn oil in both the phases. The decline in the expression of VEGF was highly marked with FO+CO(2.5∶1)+DMH in the post-initiation phase. The earlier studies have also shown that supplementation of EPA and DHA suppress the NF-κB expression in prostate cancer [47], [48]. It has also been documented that DHA, present in fish oil, blocked the angiogenesis by reducing the expression of VEGF in retinopathy [49].

In the current study, administration of different ratios of FO and CO has been used to modify the ratios of tissue n-3 and n-6 PUFAs which would subsequently alter the apoptosis by multiple pathways. n-3 PUFAs has been reported to modulate PTEN which further regulates cell death by PI3K, Akt, caspase pathway [50], [51], [13]. PTEN expression in breast cancer cell lines has also shown to significantly inhibit the phosphorylation and DNA binding activity of NF-kB and transcription of its downstream targets which include VEGF and antiapoptotic genes Bcl-2 and Bcl-xL [15]. Therefore, in the current study increasing FO consumption led to augmented PTEN expression which upregulates the apoptotic signaling by decreasing NF-kB expression and thereby, results in tumor regression.

## Conclusion

A reduction in inflammation due to PTEN modulated expression of NF- κB with EPA and DHA, major components of fish oil, and elevation in apoptosis by PI3K signaling have beneficial effect on the colonic mucosa and abrogate the progression of colon carcinogenesis. Based on the results of the current study, it can be concluded that fish oil has a dose and time dependent effect on apoptosis and several inflammatory mediators. However further studies are needed to determine the regulation of signalling molecules at the genetic level in order to use altered ratios of dietary PUFAs as an effective therapeutic approach to abrogate cancer development.
